# In Vitro Study of the Fibrinolytic Activity via Single Chain Urokinase-Type Plasminogen Activator and Molecular Docking of FGFC1

**DOI:** 10.3390/molecules26071816

**Published:** 2021-03-24

**Authors:** Chunli Gao, Quan Shen, Pengjie Tang, Yuling Cao, Houwen Lin, Bailin Li, Peng Sun, Bin Bao, Wenhui Wu

**Affiliations:** 1 College of Food Science and Technology, Shanghai Ocean University, Shanghai 201306, China; chunlgao@163.com (C.G.); quanshen863@163.com (Q.S.); 15722869010@163.com (P.T.); yuling@163.com (Y.C.); blli@shou.edu.cn (B.L.); bbao@shou.edu.cn (B.B.); 2 Research Center for Marine Drugs, State Key Laboratory of Oncogenes and Related Genes, Department of Pharmacy, Ren Ji Hospital, School of Medicine, Shanghai Jiao Tong University, Shanghai 200127, China; franklin67@126.com; 3 Shanghai Engineering Research Center of Aquatic-Product Processing and Preservation, Shanghai 201306, China; 4 School of Pharmacy, Second Military Medical University, 325 Guohe Road, Shanghai 200433, China; sunpeng78@126.com

**Keywords:** FGFC1, pro-uPA, plasminogen, molecular docking, fibrinolytic properties

## Abstract

Fungi fibrinolytic compound 1 (FGFC1) is a rare marine-derived compound that can enhance fibrinolysis both in vitro and in vivo. The fibrinolytic activity characterization of FGFC1 mediated by plasminogen (Glu-/Lys-) and a single-chain urokinase-type plasminogen activator (pro-uPA) was further evaluated. The binding sites and mode of binding between FGFC1 and plasminogen were investigated by means of a combination of in vitro experiments and molecular docking. A 2.2-fold enhancement of fibrinolytic activity was achieved at 0.096 mM FGFC1, whereas the inhibition of fibrinolytic activity occurred when the FGFC1 concentration was above 0.24 mM. The inhibition of fibrinolytic activity of FGFC1 by 6-aminohexanoic acid (EACA) and tranexamic acid (TXA) together with the docking results revealed that the lysine-binding sites (LBSs) play a crucial role in the process of FGFC1 binding to plasminogen. The action mechanism of FGFC1 binding to plasminogen was inferred, and FGFC1 was able to induce plasminogen to exhibit an open conformation by binding through the LBSs. The molecular docking results showed that docking of ligands (EACA, FGFC1) with receptors (KR1–KR5) mainly occurred through hydrophilic and hydrophobic interactions. In addition, the binding affinity values of EACA to KR1–KR5 were −5.2, −4.3, −3.7, −4.5, and −4.3 kcal/moL, respectively, and those of FGFC1 to KR1–KR5 were −7.4, −9.0, −6.3, −8.3, and −6.7 kcal/moL, respectively. The findings demonstrate that both EACA and FGFC1 bound to KR1–KR5 with moderately high affinity. This study could provide a theoretical basis for the clinical pharmacology of FGFC1 and establish a foundation for practical applications of FGFC1.

## 1. Introduction

Plasminogen is a protein with a molecular weight of 92 kDa composed of 791 amino acids, exhibiting a concentration of approximately 2 µM in total human plasma [1,2]. Plasminogen is an inactive form of plasmin, which degrades fibrin and plays a key role in the fibrinolytic system [3,4]. It is activated into plasmin as a result of cleavage between Arg561 and Val562 by tissue-type plasminogen activator (tPA) or urokinase-type plasminogen activator (uPA) [5,6]. Plasminogen adopts two conformations, i.e., closed and open. The native circulating Glu-plasminogen, which consists of seven distinct domains (a Pan-apple domain (PAp), five kringle domains (KR1–KR5, each comprising around 80 amino acid residues), and a serine protease domain (SP)), adopts a closed conformation [7,8,9]. The open conformation, which occurs through ready activation by plasminogen activators, can be formed when the kringle domains interact with fibrin or cell surface receptors [10,11,12]. Studies by Law et al. suggest that the lysine-binding sites (LBSs) of the kringle domains are essential for the interaction between plasminogen and fibrin or cell surface receptors [9,13]. 6-Aminohexanoic acid (EACA) and tranexamic acid (TXA) are lysine analogues that act as antifibrinolytic drugs. They compete with fibrin or cell surface receptors for binding to the LBSs of plasminogen, resulting in an abnormal change in plasminogen conformation, thus preventing the process of plasminogen activation [14,15]. The removal of PAp results in the formation of Lys-plasminogen. However, this is different from the open conformation, which is formed via contact with fibrin or cell surface receptors [16,17,18].

Fungi fibrinolytic compound 1 (FGFC1, 2,5-bis-[8-(4,8-dimethyl-nona-3,7-dienyl)-5,7-dihydroxy-8-methyl-3-keto-1,2,7,8-tertahydro-6*H*-pyran[a]isoindol-2-yl]-pentanoic acid), is a rare compound with a molecular weight of 869 Da isolated from the culture of marine fungi *Stachybotrys longispora* FG216 (CCTCCM 2012272). FGFC1 is a thrombolytic agent without hemorrhagic risk that can degrade fibrin both in vitro and in vivo. The pharmacokinetics of FGFC1 in Wistar rats indicated that FGFC1 becomes quickly distributed in most tissues but is not distributed in the brain. FGFC1 (10 mg/mL) could dissolve most of the pulmonary thrombi when used to treat Wistar rats. FGFC1 is a potential drug for the treatment of thrombosis [19,20,21]. Furthermore, FGFC1 possesses fibrinolytic activity mainly through influencing the secondary and tertiary structure of plasminogen, enabling its activation [22]. Accordingly, the key target of FGFC1 is plasminogen. The enzyme kinetic parameters of FGFC1 were also determined [23]. However, the fibrinolytic characterization of FGFC1 requires further research. Currently, there remain questions to be answered. Which sites of plasminogen bind to FGFC1? How does the conformation of plasminogen change after binding to FGFC1? EACA inhibits Plg binding by competing with fibrin or cell surface receptors for the lysine-binding sites (LBSs). According to Takayasu et al., the levels of fibrin binding of ^125^I-Glu-Plg and ^125^I-Lys-Plg were increased by staplabin at the same concentration. In both cases, binding was inhibited by EACA, suggesting that the activation process of plasminogen involves LBSs [24,25,26]. On the basis of the above results, we aimed to measure the fibrinolytic activity of FGFC1 in the presence of EACA and TXA. Molecular docking is a powerful tool to predict the binding mode and binding sites between a protein and a small molecule. AutoDock Vina is a molecular docking program for protein–ligand docking with high accuracy and computation speed. AutoDock Vina computes the best conformation and placement of ligands. Ligands are ranked on the basis of their binding ability [27,28]. The determination of binding sites and the binding mode between FGFC1 and plasminogen is important for exploring the mechanism of their interaction. 

In this study, we constructed a fibrinolytic system composed of plasminogen (PLG) and pro-uPA in vitro on the basis of previous studies to evaluate the fibrinolytic characterization of FGFC1 mediated by plasminogen and pro-uPA. In addition, the fibrinolytic system was used to investigate the binding sites and mode of binding between FGFC1 and plasminogen, thereby inferring the mechanism of their interaction. These findings are significant for studying the in vivo pharmacology of FGFC1.

## 2. Results

### 2.1. Fibrinolytic Characterization of FGFC1

#### 2.1.1. The Role of Pro-uPA in the Fibrinolytic Activity of FGFC1

**Figure 1:**
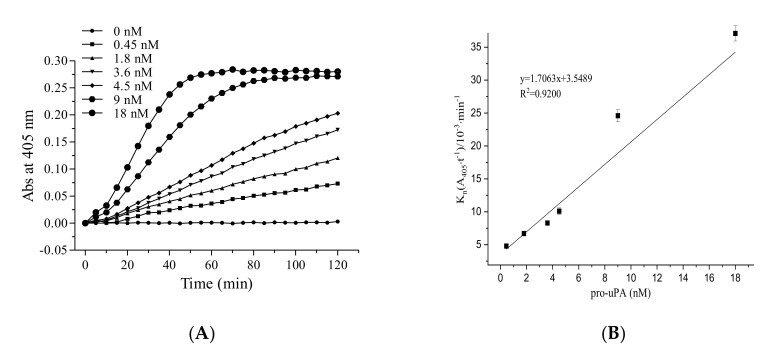
The role of the single-chain urokinase-type plasminogen activator (pro-uPA) in the fibrinolytic activity of fungi fibrinolytic compound 1 (FGFC1). Plot of absorbance at 405 nm (A405) versus time (**A**) and the values of K_n_ (**B**) at different pro-uPA concentrations (0–18 nM). The reaction was incubated at 37 °C and measured every 5 min for 120 min. The results are expressed as the mean ± SD performed in triplicate. The concentration of Glu-plasminogen and FGFC1 in the reaction system were 40 nM and 0.048 mM, respectively.

In general, fibrinolytic activity reached a maximum between 20 and 40 min (Figure 1A). The fibrinolytic activity increased approximately linearly with pro-uPA concentration, showing 7.7-fold enhancement (4.8 × 10^−3^ to 37.1 × 10^−3^ min^−1^) from 0.45 to 18 nM (Figure 1B). The addition of pro-uPA promoted the conversion of plasminogen to plasmin; additionally, more pro-uPA was activated to uPA with the increase in plasmin.

#### 2.1.2. The Role of Glu-Plasminogen in the Fibrinolytic Activity of FGFC1

**Figure 2:**
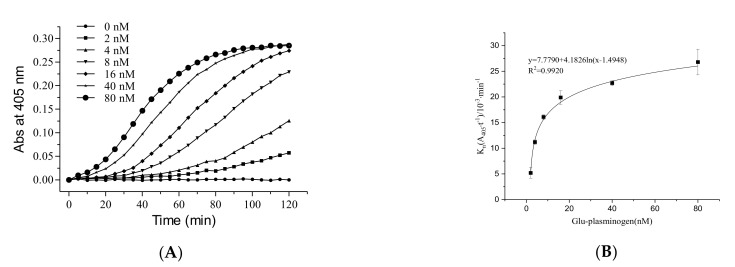
The role of Glu-plasminogen in the fibrinolytic activity of FGFC1. Plot of A_405_ versus time (**A**) and the values of K_n_ (**B**) at different Glu-plasminogen concentrations (0–80 nM). The reaction was incubated at 37 °C and measured every five minutes for 120 min. The results are expressed as the mean ± SD, performed in triplicate. The concentration of pro-uPA and FGFC1 in the reaction system were 9 nM and 0.018 mM, respectively.

The time required to achieve maximum reaction rate was shortened as the Glu-plasminogen concentration increased (Figure 2A). From 2 to 80 nM, Glu-plasminogen promoted the fibrinolytic activity in a dose-dependent manner until saturation was reached. The fibrinolytic activity was enhanced 5.2-fold (5.2 × 10^−3^ to 26.8 × 10^−3^ min^−1^) with an increase in Glu-plasminogen concentration from 2 to 80 nM (Figure 2B). At high concentrations of Glu-plasminogen, there was increased activation of plasmin, which then accelerated the conversion of pro-uPA to uPA. However, this effect stabilized at Glu-plasminogen concentrations higher than 16 nM.

#### 2.1.3. The Fibrinolytic Characterization of FGFC1 Mediated by Pro-uPA and Glu-Plasminogen

**Figure 3:**
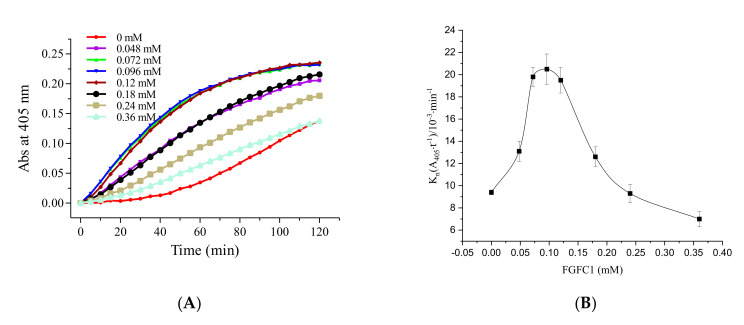
The fibrinolytic characterization of FGFC1 mediated by pro-uPA and Glu-plasminogen. Plot of A_405_ versus time (**A**) and the values of K_n_ (**B**) at different FGFC1 concentrations (0–0.36 mM). The reaction was incubated at 37 °C and measured every five min for 120 min. The results are expressed as the mean ± SD, performed in triplicate. The concentration of pro-uPA and Glu-plasminogen in the reaction system were 9 nM and 4 nM, respectively.

As shown in Figure 3, at concentrations of FGFC1 up to 0.24 mM, FGFC1 promoted fibrinolytic activity; however, any further increase in concentration had no effect. In particular, when the FGFC1 concentration was below 0.096 mM, fibrinolytic activity was enhanced by FGFC1 in a dose-dependent manner. From 0.096 to 0.24 mM, the promoting effect of FGFC1 declined in a dose-dependent manner. The fibrinolytic activity reached its maximum and showed a 2.2-fold enhancement at 0.096 mM (Figure 3B).

The data indicate that the effect of FGFC1 on Glu-plasminogen was divided into two aspects—activation and inhibition. A relatively low FGFC1 concentration (<0.096 mM) facilitated the ready activation of Glu-plasminogen to plasmin. However, beyond this concentration, excess FGFC1 potentially acts as a Glu-plasminogen inhibitor. As the FGFC1 concentration increased beyond 0.24 mM, the inhibitory action of FGFC1 became dominant, thereby preventing fibrinolytic activity.

#### 2.1.4. The Fibrinolytic Characterization of FGFC1 Mediated by Pro-uPA and Lys-Plasminogen

**Figure 4:**
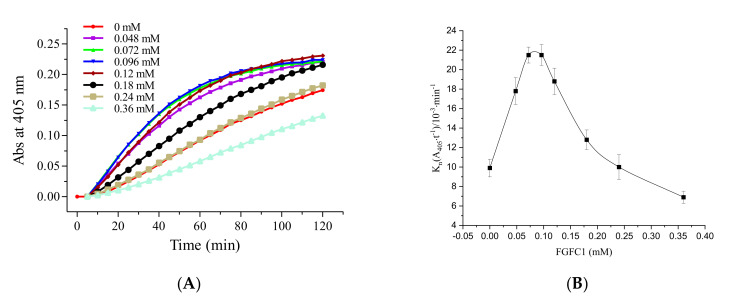
The fibrinolytic characterization of FGFC1 mediated by pro-uPA and Lys-plasminogen. Plot of A_405_ versus time (**A**) and the values of Kn (**B**) at different FGFC1 concentrations (0–0.36 mM). The reaction was incubated at 37 °C and measured every five minutes for 120 min. The results are expressed as the mean ± SD, performed in triplicate. The concentration of pro-uPA and Lys-plasminogen in the reaction system were 9 and 4 nM, respectively.

To investigate whether the effect of FGFC1 on Glu-plasminogen resulted in a conversion of Glu-plasminogen to Lys-plasminogen, we substituted Glu-plasminogen with Lys-plasminogen to measure the fibrinolytic activity of FGFC1. As shown in Figure 4, FGFC1 promoted fibrinolytic activity even when mediated by Lys-plasminogen. A concentration of 0.24 mM still distinguished between activation and inhibition of fibrinolytic activity. The fibrinolytic activity of FGFC1 was again separable into two phases. When the FGFC1 concentration was below 0.072 mM, fibrinolytic activity was enhanced in a dose-dependent manner. From 0.096 to 0.24 mM, the promoting effect of FGFC1 declined in a dose-dependent manner. The fibrinolytic activity also showed a 2.2-fold increase at 0.072 mM (Figure 4B).

Compared with Glu-plasminogen, the fibrinolytic activity mediated by Lys-plasminogen was stronger when the FGFC1 concentration was below 0.048 mM, and reached its maximum activity at approximately 0.072 mM. Plasmin generation was higher with lys-plasminogen as would be expected and a similar trend was observed in the fibrinolytic activity of FGFC1 with both Glu- and Lys-plasminogen. These data show that FGFC1 at a particular range of concentrations may be used to stimulate the conversion of Glu-plasminogen to Lys-plasminogen. 

### 2.2. Binding Sites and Mode of Binding between FGFC1 and Plasminogen

#### 2.2.1. The Effect of EACA, TXA, and Soybean Trypsin Inhibitor (SBTI) on the Fibrinolytic Activity of FGFC1 

As shown in Table 1, the fibrinolytic activity of FGFC1 was promoted within a certain concentration range (0–0.18 mM), reaching its peak at 0.096 mM. However, at concentrations higher than 0.18 mM, the addition of EACA, TXA, and SBTI inhibited the fibrinolytic activity. EACA (3.6–216 mM) and TXA (0.72–21.6 mM) remarkably inhibited the fibrinolytic activity of FGFC1, thus confirming that FGFC1 enhances fibrinolytic activity through binding of plasminogen. The activation of plasminogen by FGFC1 was inhibited by EACA and TXA, strongly indicating the participation of LBSs. This occurred in a dose-dependent manner for concentrations of EACA and TXA ranging from 3.6 to 21.6 mM and from 0.72 to 4.8 mM, respectively, with the effect stabilizing beyond these ranges. The half maximal inhibitory concentration (IC_50_) values of EACA and TXA were approximately 9.6 and 1.44 mM, respectively.

**Figure 5:**
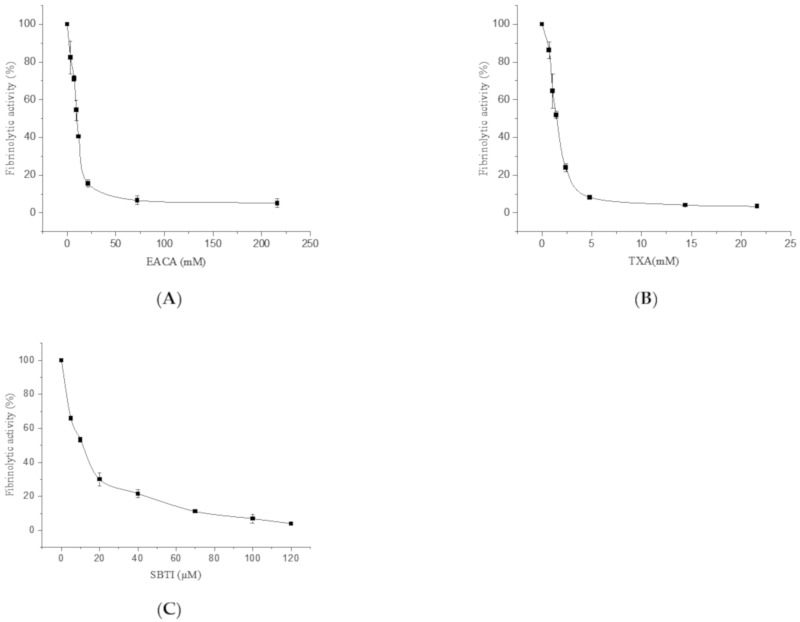
The fibrinolytic activity of FGFC1 at different concentrations of EACA (0–216 mM) (**A**), TXA (0–21.6 mM) (**B**), and SBTI (0–120 µM) (**C**). The reaction was incubated at 37 °C and measured at 405 nm using a microplate reader every five minutes for 120 min. Results are expressed as a percentage of the fibrinolytic activity in the absence of EACA, TXA, and SBTI. The results are expressed as the mean ± SD, performed in triplicate. The concentration of FGFC1 for the FGFC1 + EACA, FGFC1 + TXA, and FGFC1 + SBTI assays was 0.12 mM.

EACA and TXA competed with FGFC1 for the LBSs of the kringle domains, blocking the binding between FGFC1 and LBS, thereby inhibiting the activation of plasminogen. EACA and TXA partially bound to LBSs at low concentrations (<21.6 and <4.8 mM, respectively), which was consistent with the incomplete inhibition of the fibrinolytic activity of FGFC1. When the concentrations of EACA and TXA were beyond 72 and 14.4 mM, respectively, the LBSs were almost completely occupied; therefore, the fibrinolytic activity of FGFC1 was almost completely inhibited (Figure 5A,B). In addition, we measured fibrinolytic activity in the presence of SBTI (trypsin inhibitor from *Glycine max*) to test its inhibitory effect. As shown in Table 1, its inhibitory effect (5–120 mM) was relatively stronger than that of EACA and TXA. When the concentration of SBTI ranged from 5 to 70 mM, the fibrinolytic activity of FGFC1 was inhibited in a dose-dependent manner, and stabilized beyond this point (Table 1). The results show that SBTI inhibits the activity of plasmin, resulting in a decrease in the fibrinolytic activity of FGFC1. The inhibitory effect of SBTI was 1000 times stronger than that of EACA (Figure 5C). 

#### 2.2.2. Docking

To obtain more insight into the action mechanism of FGFC1’s interaction with the five kringle domains (KR1–KR5), the docking of two ligands (EACA and FGFC1) with KR1–KR5 was simulated. The results of EACA binding to KR1–KR5 were concordant with previous NMR and X-ray diffraction experiments, suggesting that the parameters used for AutoDock Vina calculations were appropriate [29,30,31,32]. 

**Figure 6:**
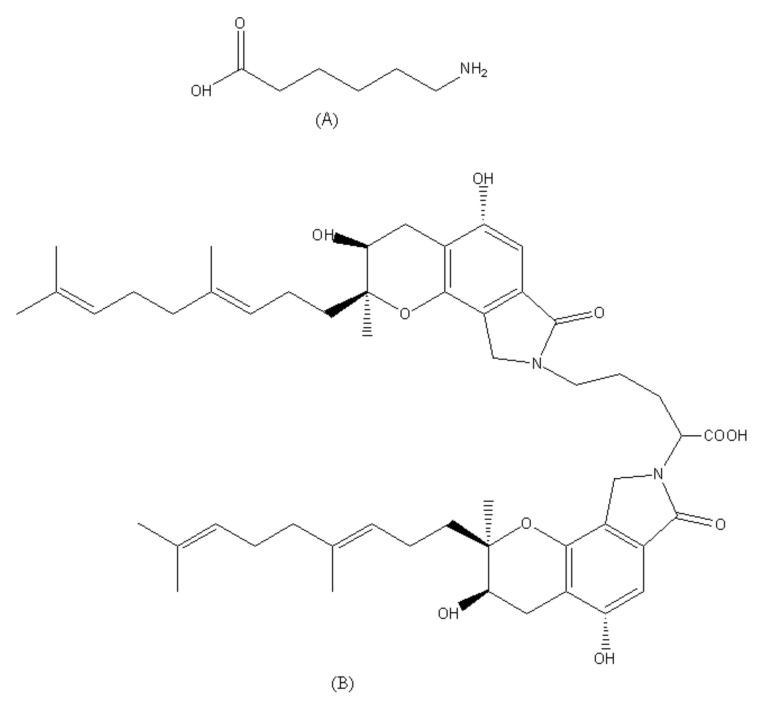
Chemical structures of EACA (**A**) and FGFC1 (**B**)**.**

The chemical structures of EACA and FGFC1 are shown in Figure 6. 

**Figure 7:**
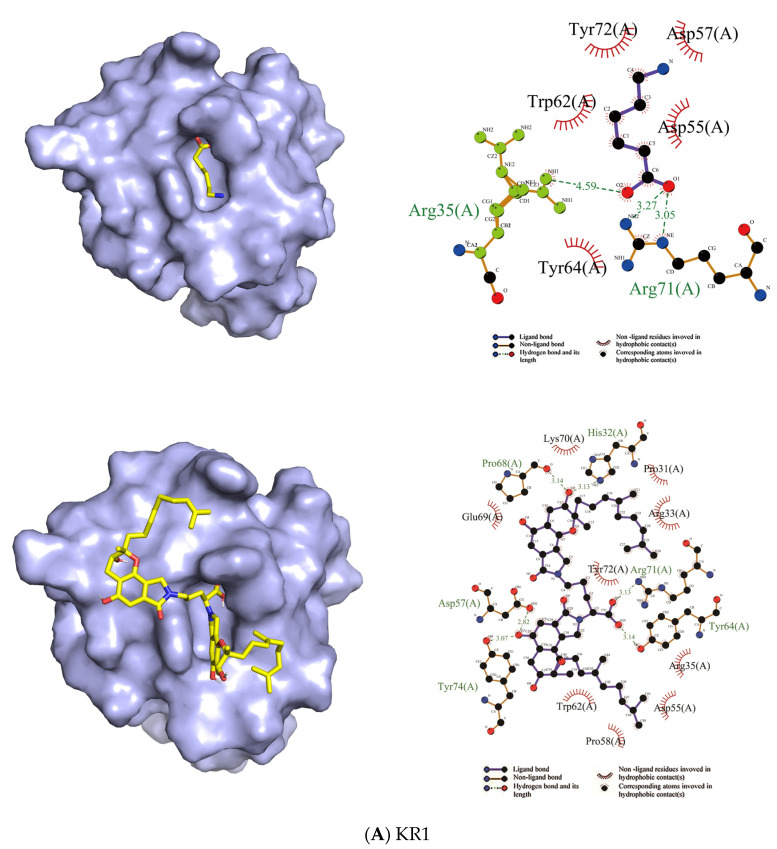

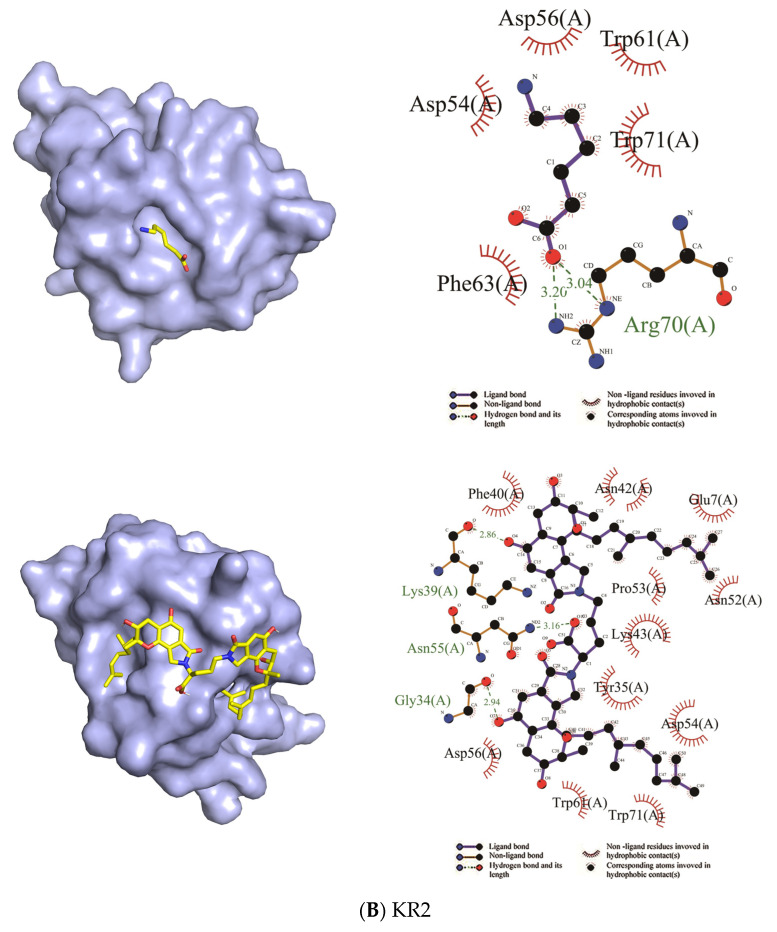

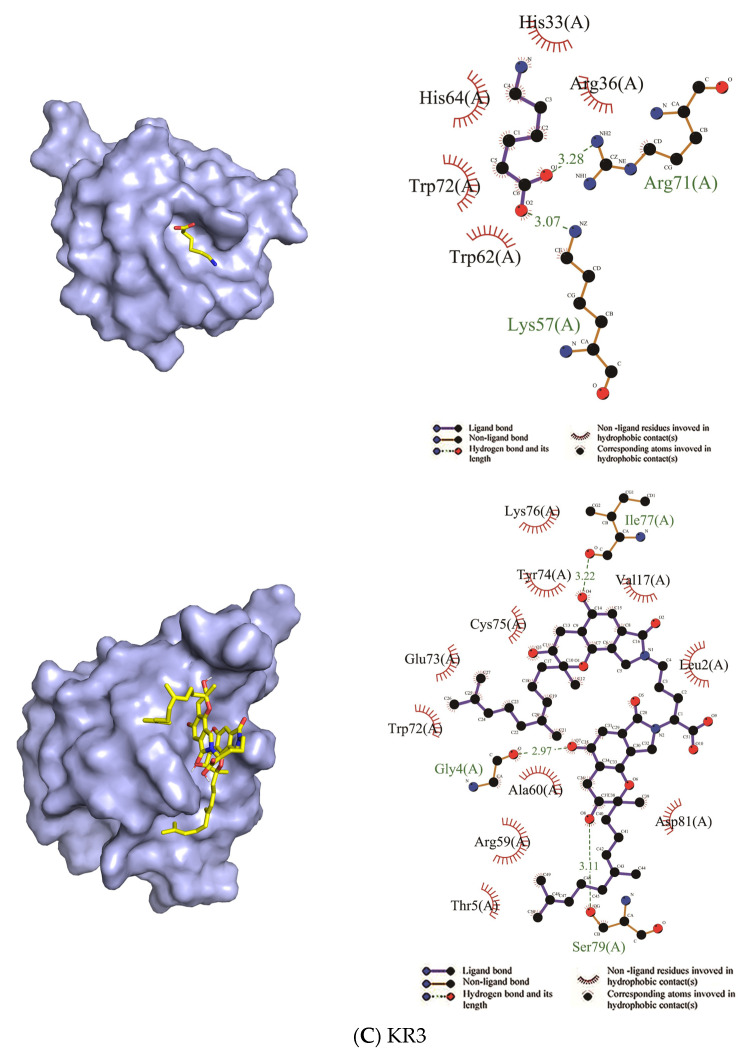

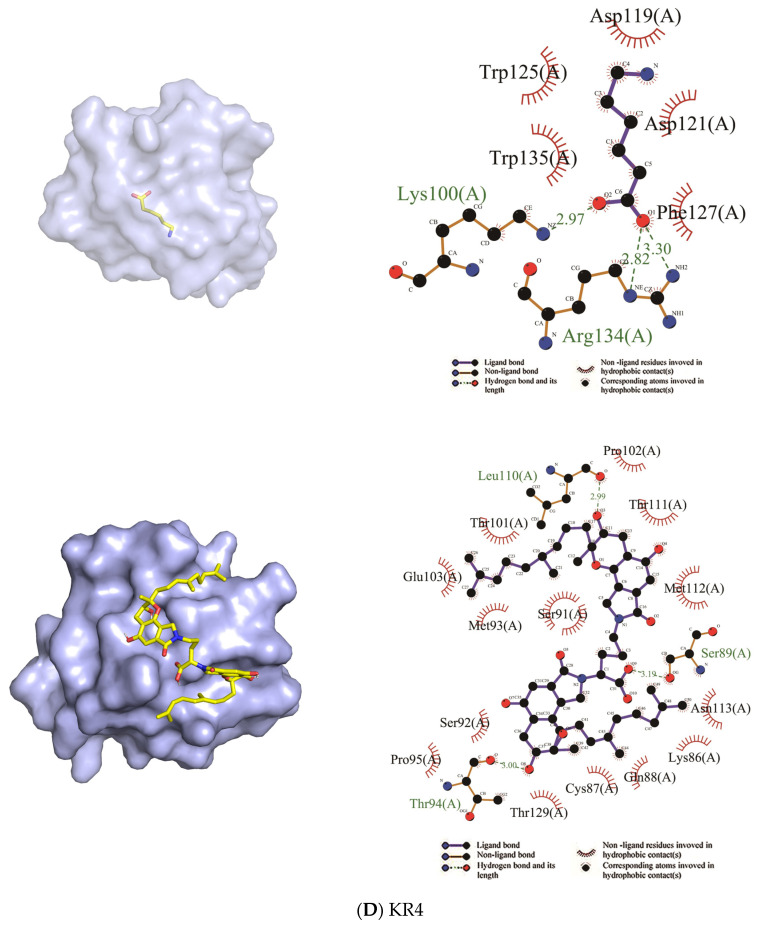

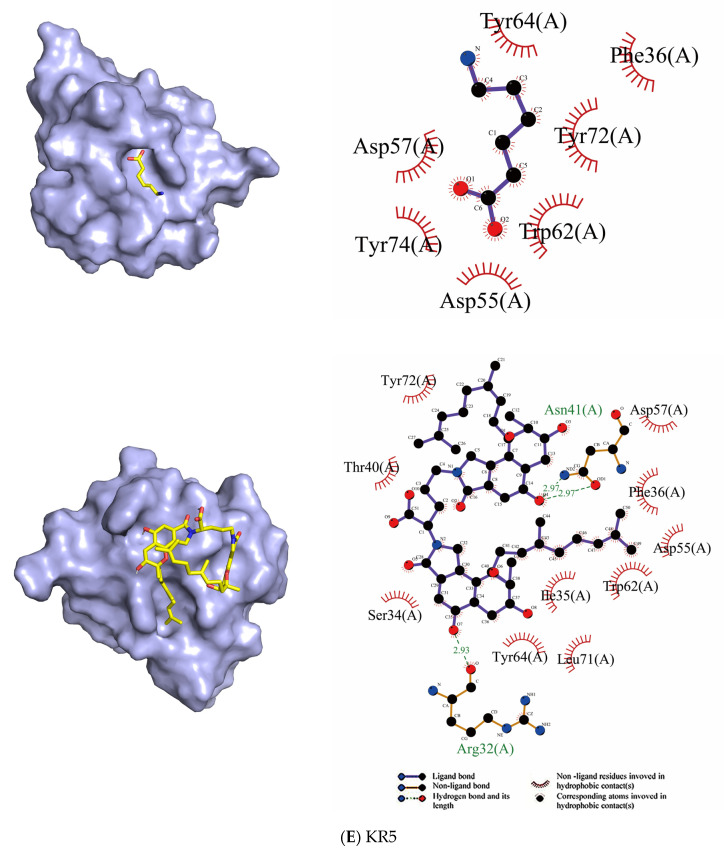
The docking results of ligands (EACA and FGFC1) with kringle domains (KR1–KR5) (**A**–**E**). In each group, the docking results of EACA (top) and FGFC1 (bottom) with KR1–KR5 are shown, along with the binding site (left) and amino acid residues involved (right). The dotted lines represent hydrogen bonds between the ligand and amino acid residues. KR1–KR5 are shown in surface representation, whereas EACA and FGFC1 are shown as sticks.

The same protein orientation was used to compare the binding models of EACA and FGFC1 to the same kringle domain. As shown in Figure 7, the binding position of FGFC1 to KR1–KR5 partially overlapped with that of EACA according to a comparison and analysis of their binding modes. 

As shown in Figure 7 and Table 2, the molecular docking results identified hydrogen bonding and hydrophobic interactions between the ligands (EACA, FGFC1) and receptors (KR1–KR5). Two hydrogen bonds (3.05 and 3.27 Å) were formed between EACA and residue Arg71 of KR1, in addition to strong hydrophobic interactions with residues Tyr64, Asp55, Asp57, Tyr72, and Trp62. FGFC1 formed hydrogen bonds with residues Tyr64, Arg71, His32, Pro68, Asp57, and Tyr74 of KR1, with bond lengths of 3.14, 3.13, 3.13, 3.14, 2.82, and 3.07 Å, respectively (Figure 7A). 

Two hydrogen bonds were formed between EACA and Arg70 of KR2 (3.20 and 3.04 Å) in addition to hydrophobic interactions with Trp71, Trp61, Asp56, Asp54, and Phe63. FGFC1 formed hydrogen bonds with residues Gly34, Asn55, and Lys39 of KR2, with bond lengths of 2.94, 3.16, and 2.86 Å, respectively, along with strong hydrophobic interactions involving Trp71, Asp54, Tyr35, Lys43, Pro53, Asn52, Glu7, Asn42, Phe40, Asp56, and Trp61 (Figure 7B). 

EACA formed two hydrogen bonds (3.07 Å and 3.28 Å) with Lys57 and Arg71 of KR3, respectively, in addition to strong hydrophobic interactions with Arg36, His33, His64, Trp72, and Trp62. FGFC1 formed three hydrogen bonds (3.11, 3.22, and 2.97 Å) with residues Ser79, Ile77, and Gly4 of KR3, in addition to hydrophobic interactions with Asp81, Leu2, Val17, Lys76, Tyr74, Cys75, Glu73, Trp72, Ala60, Arg59, and Thr5 (Figure 7C). 

EACA formed three hydrogen bonds with Lys100 (2.97 Å) and Arg134 (2.82 and 3.30 Å) of KR4 in addition to hydrophobic interactions with Trp135, Trp125, Asp119, Asp121, and Phe127. Three hydrogen bonds were formed between FGFC1 and Thr94, Ser89, and Leu110 of KR4, with bond lengths of 3.00, 3.19, and 2.99 Å, respectively, in addition to strong hydrophobic interactions with Ser92, Pro95, Thr129, Cys87, Gln88, Lys86, Asn113, Met112, Thr111, Pro102, Thr101, Glu103, Met93, and Ser91 (Figure 7D). 

EACA exhibited strong hydrophobic interactions with residues Tyr74, Asp55, Trp62, Tyr72, Phe36, Tyr64, and Asp57 of KR5. FGFC1 formed hydrogen bonds with residues Asn41 and Arg32 of KR5 with bond lengths of 2.97 and 2.93 Å, respectively, in addition to strong hydrophobic interactions with Tr64, Leu71, Ile35, Trp62, Asp55, Phe36, Asp57, Tyr72, Thr40, and Ser34 (Figure 7E).

The analysis results show that FGFC1 and EACA were located in the pocket of the five kringle domains. According to the analysis of the binding modes, hydrogen bonds and hydrophobic carbon–hydrogen interactions played a significant role in the binding of both FGFC1 and EACA to KR1–KR5. 

The binding affinity values for the most appropriate binding modes are shown in Table 3. The values obtained for EACA to KR1–KR5 were −5.2, −4.3, −3.7, −4.5, and −4.3 kcal/mol, respectively, whereas those for FGFC1 to KR1–KR5 were −7.4, −9.0, −6.3, −8.3, and −6.7 kcal/moL, respectively. The docking results showed that both EACA and FGFC1 bound to KR1–KR5 with moderately high binding affinity. In general, the binding affinity of FGFC1 to KR1–KR5 was stronger than that of EACA. These results may help to explain observations in the previous experiment whereby fibrinolytic activity was enhanced for equal concentrations of FGFC1 and EACA. The binding affinity of FGFC1 to KR2 and EACA to KR1 was strongest, suggesting that KR2 and KR1, respectively, are central to their activation of plasminogen. 

**Figure 8:**
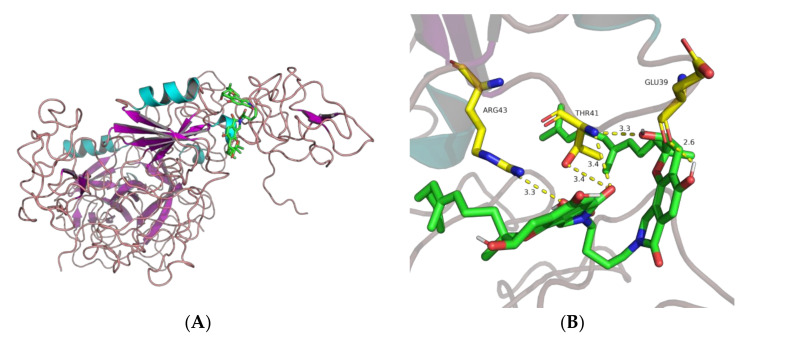
Schematic diagram of optimal conformational interaction between FGFC1 and plasminogen. (**A**) Binding site and (**B**) the 3D model of FGFC1 docking with protein.

The present study also investigated the interaction model between the proteins and compounds at the molecular level. Compound FGFC1 was conjugated to plasminogen with a binding affinity of −8.0 kcal/moL, and the theoretical binding mode is shown in Figure 8. 

It can be seen from Figure 8 that the active pocket of compound FGFC1 presented a compact binding mode. FGFC1 formed hydrogen bonds with amino acid residues Glu39 (2.6 Å), Thr41 (3.3 and 3.4 Å), and Arg43 (3.4 Å). These interactions gave rise to a stable complex.

## 3. Discussion

In the present work, we constructed the fibrinolytic system and characterized the fibrinolytic activity of FGFC1. The binding sites and mode of binding between FGFC1 and plasminogen were investigated using a combination of experiments and bioinformatic docking. It was first discovered that a relatively low FGFC1 concentration enhances fibrinolytic activity, whereas excess FGFC1 is inhibitory in vitro, in contrast with results of previous research [21,23]. These findings are instructive for the use of FGFC1 in vivo. 

The conformation of Glu-plasminogen (Glu-Plg) differs from that of Lys-plasminogen (Lys-Plg), and Glu-Plg has a closed conformation. In intact plasminogen, cleavage between Lys77 and Lys78 results in the removal of the PAp domain. Lys-Plg lacks the PAp domain and, as such, has an open conformation. Compared with the Glu-Plg-mediated fibrinolytic activity of FGFC1, the Lys-Plg-mediated fibrinolytic activity reached its maximum at a lower concentration of FGFC1 (approximately 0.072 mm) (Figure 4). The results show that different conformations led to different activity. This may have been due to the different conformations resulting in different docking sites. The activation mechanism of FGFC1 to Glu-plasminogen may involve the conversion of Glu-plasminogen to Lys-plasminogen. The docking results of FGFC1 and KR1–KR5 show that the participation of LBSs is essential for the activation of plasminogen by FGFC1. Only the LBS of KR1 is unprotected, and KR1 mediates the initial interaction between plasminogen and the C-terminal lysine moiety on the cell surface or fibrin. KR5 peeling away from the PAp may be transiently exposed in closed plasminogen; hence, it can interact with an external lysine, triggering a conformational change. Notably, Lys50 of the PAp domain interacts with Asp518 of KR5, forming a strong salt bridge, which is a key interaction. Arg70 of the PAp domain coordinates KR5 via interaction with Asp534 [9,13,14,33]. Surprisingly, our results for docking between FGFC1 and KR5 showed that the group of FGFC1 interacted with Asp518 and Asp534, forming hydrogen bonds (Figure 7E). The results of molecular docking between FGFC1 and plasminogen showed that FGFC1 formed stable complexes via hydrogen bonds with Thr41, Glu39, and Arg43. This was consistent with the docking between FGFC1 and KR5, which formed hydrogen bonds through interactions between the group of FGFC1 and Asp (Figure 8). The docking results show that the conformation of plasminogen may be changed.

**Figure 9:**
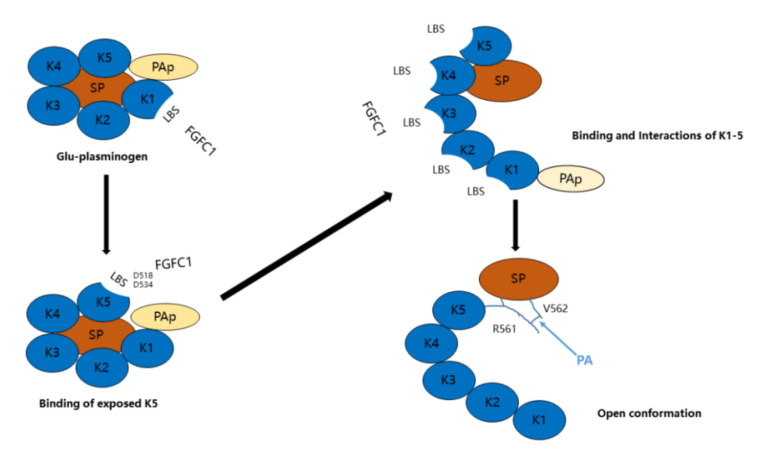
A model of the mechanism of plasminogen activation by FGFC1.

The mechanism of FGFC1–plasminogen interaction was inferred from a comprehensive analysis of previous studies and current results. The main points are described below. When the concentration of FGFC1 is relatively low, the function of FGFC1 may mimic the cell surface or fibrin. The mechanism of plasminogen activation by FGFC1 is shown in Figure 9. FGFC1 initially binds to the LBS of KR1, resulting in the peeling of KR5 from PAp and its exposure in closed plasminogen. Subsequently, FGFC1 mainly interacts with Asp518 and Asp534 of the exposed KR5, triggering a conformational change and structural rearrangement. At this moment, the additional LBSs of the kringle domains are exposed, allowing the PAp domain to move. Thus, FGFC1 can bind to additional LBSs, and a series of interactions occur with the kringle domains, leading to the formation of an open conformation that is readily activated by plasminogen activators. By contrast, when the concentration of FGFC1 is relatively high, FGFC1 may functionally mimic EACA or TXA due to having similar functional groups (carboxyl group and Nɛ sidechain) and binding sites for plasminogen. Excess FGFC1 may bind specific sites of plasminogen, resulting in the formation of an abnormal conformation, which inhibits the activation of plasminogen.

The complete plasminogen consists of seven different domains (a Pan-apple domain (PAp), five cyclic domains (KR1–KR5), and a serine protease domain (SP)). Serine protease plays an important role in digestion, coagulation, and the complement system, and is mainly regulated by protease inhibitors. Serine protease inhibitors (serpins) can change from a natural form to a polymorphic type, showing extensive flexibility. Research findings that the reactive site and β-sheet polymorphism appear to be coupled in serpins may account for the extreme stability of serpin–proteinase complexes through the insertion of the reactive site strand into a β-sheet [34]. In this study, we confirmed the existence of polymorphism via molecular docking and fibrinolytic activity. In conclusion, the mechanism of the interaction between FGFC1 and Glu-plasminogen may be related to its conversion to Lys-plasminogen and the existence of polymorphism. FGFC1 interacted with the LBSs in plasminogen via hydrogen bonds. Overall, this study provides a theoretical basis for the clinical pharmacology of FGFC1 and a reference for the development of novel plasminogen activators.

## 4. Materials and Methods

### 4.1. Materials

Glu-plasminogen, Lys-plasminogen, single-chain urokinase-type plasminogen activator (pro-uPA), trypsin inhibitor from *Glycine max* (SBTI), 6-aminohexanoic acid (EACA), tranexamic acid (TXA), and bovine serum albumin (BSA) were purchased from Sigma-Aldrich (Shanghai, China). The chromogenic substrate S-2444 for urokinase-type plasminogen activator (uPA) was purchased from BioMed (Shanghai, China). Absorbance was measured using a microplate reader (SH-1000 Lab, Corona Electric, Ibaraki, Japan). Fungi fibrinolytic compound 1 (FGFC1) was extracted and purified in our laboratory (purity > 98%). All other chemicals were of analytical grade.

### 4.2. Fibrinolytic Characterization of FGFC1

In this experiment, the fibrinolytic activity of FGFC1 was expressed as K_n_ (the slope of each curve) and determined by the linear regression between absorbance and time. The content of uPA was represented by the absorbance of hydrolytic product *p*-nitroaniline (p-NA) in S-2444 at 405 nm. The fibrinolytic reaction system was carried out as previously described with slight modifications on the basis of the following feedback activation reactions [21,35,36]:

(Reaction 1): plasminogen activated to plasmin by pro-uPA;
(Reaction 2): pro-uPA activated to uPA by plasmin;
(Reaction 3): substrate S-2444 hydrolyzed by uPA.

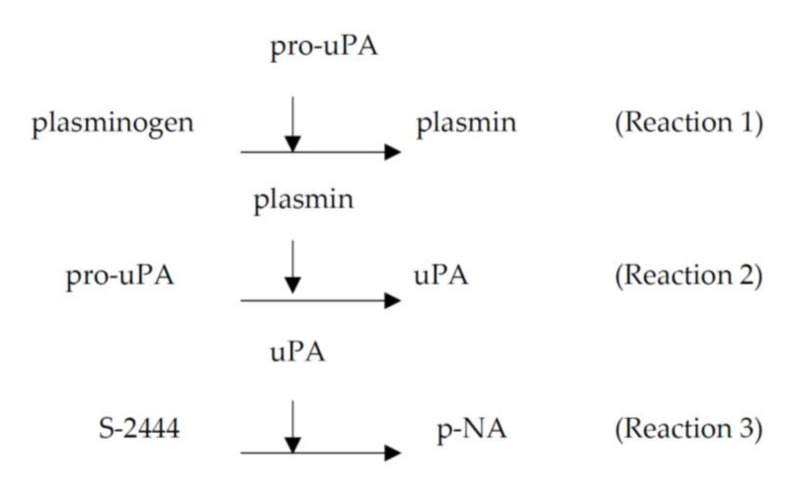

#### 4.2.1. The Role of Pro-uPA in the Fibrinolytic Activity of FGFC1

The reaction mixture contained pro-uPA (0.45, 1.8, 3.6, 4.5, 9.0, and 18 nM), Glu-plasminogen (40 nM), FGFC1 (0.048 mM), S-2444 (0.4 mM), BSA (10 mg/mL), and Tris-HCl buffer (50 mM, 100 mM NaCl, pH 7.4 at 25 °C). The reaction mixture was measured in a 96-well plate at 37 °C for 120 min. The time–absorbance curves were determined at 405 nm using a microplate reader. The fibrinolytic activity of different pro-uPA concentrations was obtained from the time–absorbance curves.

#### 4.2.2. The Role of Glu-Plasminogen in the Fibrinolytic Activity of FGFC1

The reaction mixture contained Glu-plasminogen (2, 4, 8, 16, 40, and 80 nM), pro-uPA (9 nM), FGFC1 (0.018 mM), S-2444 (0.4 mM), BSA (10 mg/mL), and Tris-HCl buffer (50 mM, 100 mM NaCl, pH 7.4 at 25 °C). The reaction mixture was measured in a 96-well plate at 37 °C for 120 min. The time–absorbance curves were determined at 405 nm using a microplate reader. The fibrinolytic activity of different Glu-plasminogen concentrations was obtained from the time–absorbance curves.

#### 4.2.3. The FGFC1 Fibrinolytic Characterization by Pro-uPA and Glu-/Lys-Plasminogen

The reaction mixture contained FGFC1 (0.048–0.36 mM), Glu-plasminogen (4 nM), or Lys-plasminogen (4 nM), pro-uPA (9 nM), S-2444 (0.4 mM), BSA (10 mg/mL), and Tris-HCl buffer (50 mM, 100 mM NaCl, pH 7.4 at 25 °C). The reaction mixture was incubated in a 96-well plate at 37 °C for 120 min. The time–absorbance curves were determined at 405 nm using a microplate reader. The fibrinolytic activity of different FGFC1 concentrations was obtained from the time–absorbance curves.

### 4.3. The Binding Sites and Binding Mode between FGFC1 and Plasminogen

#### 4.3.1. The Effect of EACA, TXA, and SBTI on the Fibrinolytic Activity of FGFC1

FGFC1 (0.12 mM), pro-uPA (9 nM), Glu-plasminogen (4 nM), S-2444 (0.4 mM), BSA (10 mg/mL), and Tris-HCl buffer (50 mM, 100 mM NaCl, pH 7.4 at 25 °C) were incubated in the absence and presence of EACA, TXA, and SBTI. The concentrations of EACA, TXA, and SBTI ranged from 3.6 to 216 mM, 0.72 to 21.6 mM, and 5 to 120 µM, respectively. All reaction mixtures were incubated in a 96-well plate at 37 °C for 120 min. The time–absorbance curves were determined at 405 nm using a microplate reader (SH-1000 Lab, Corona Electric, Ibaraki, Japan). The fibrinolytic activity of FGFC1 in the presence/absence of EACA, TXA, and SBTI was obtained from the time–absorbance curves.

#### 4.3.2. Docking

Models of bound zwitterionic form of EACA and carboxylate form of FGFC1 to receptors (KR1–KR5, plasminogen protein) were calculated using AutoDock Vina 1.1.2. Structures for KR1–KR5 were obtained from the Protein Data Bank (PDB), with codes 4CIK, 6DCM, 2L0S, 1KRN, and 2KNF, respectively. The three-dimensional structures of EACA and plasminogen protein were downloaded from ZINC database (http://zinc.docking.org/ (accessed on 24 March 2021)) and the Research Collaboration for Structural Bioinformatics (RCSB) PDB (www.rcsb.org (accessed on 24 March 2021)), respectively, and the three-dimensional structure of FGFC1 was completed using ChemOffice Professional 19. The small molecules EACA and FGFC1 were modeled as zwitterions and carboxylate respectively with ChemOffice Professional 19. 

The molecular docking steps are described below.

Step 1. Ligand Pretreatment

Energy minimization of the small molecules were done using ChemBio3D Ultra 14.0 and saved in mol2 format. Further, the small-molecule compounds EACA and FGFC1 were selected as ligands after adding hydrogen, calculating charges, and distributing charges with Autodock Tools 1.5.6. Then, the rotatable key of ligands was set before saving them in pdbqt format using Autodock Tools 1.5.6.

Step 2. Receptor Pretreatment

For receptor preparation, PyMOL software was used to remove original ligand and all water molecules of the protein molecule. Then, the acceptor was defined as the receptor and saved in pdbqt format after adding hydrogens, calculating charges, and distributing charges using Autodock Tools 1.5.6.

Step 3. Preparation of Docking Parameters

Small molecules were imported into Autodock Tools 1.5.6 to set the size of the box. The original protein ligand was used as the center of the docking box. In the absence of an original ligand, the whole protein was used as the docking area. The size of the lattice box was set to 80 × 80 × 80 (with the spacing of each lattice equal to 0.375 Å). In order to increase the accuracy of calculation, we set the exhaustiveness parameter to 20.

Step 4. Running Molecular Docking and Output Results

In this project, Autodock Vina 1.1.2 was used for semiflexible docking, and nine conformations were generated. The conformation with the best binding affinity was selected as the final docking pose.

Step 5. Analysis of Docking Results for EACA and FGFC1 to KR1–KR5

The conformation with the lowest binding energy was selected for docking mode analysis. The docking results were visualized using Ligplot and PyMOL.

Note: In the above molecular docking method, receptors (KR1–KR5, plasminogen protein) and FGFC1 were regarded as grid and flexible, respectively. There were a variety of torsions in sampling for FGFC1, so this semiflexible docking protocol had limitations.

## Figures and Tables

**Table 1:** The effect of different 6-aminohexanoic acid (EACA), tranexamic acid (TXA), and soybean trypsin inhibitor (SBTI) concentrations on the fibrinolytic activity of FGFC1.

FGFC1		FGFC1 + EACA		FGFC1 + TXA		FGFC1 + SBTI	
Concentration (mM)	Fibrinolytic Activity (%)	Concentration of EACA (mM)	Fibrinolytic Activity (%)	Concentration of TXA (mM)	Fibrinolytic Activity (%)	Concentration of SBTI (mM)	Fibrinolytic Activity (%)
0	100	0	100	0	100	0	100
0.048	139.36 ± 4.6	3.6	82.35 ± 8.7	0.72	86.19 ± 4.5	5	65.83 ± 1.2
0.072	210.64 ± 4.3	7.2	71.32 ± 1.5	1.08	64.55 ± 8.9	10	53.24 ± 1.5
0.096	218.09 ± 6.8	9.6	54.41 ± 5.4	1.44	51.87 ± 1.9	20	29.86 ± 3.9
0.12	207.45 ± 5.8	12	40.44 ± 0.1	2.4	23.88 ± 2.1	40	21.58 ± 2.3
0.18	134.04 ± 4.5	21.6	15.44 ± 1.9	4.8	8.21 ± 0.5	70	11.15 ± 0.6
0.24	98.94 ± 4.1	72	6.62 ± 2.3	14.4	4.10 ± 0.6	100	6.83 ± 2.6
0.36	74.47 ± 3.4	216	5.15 ± 2.5	21.6	3.36 ± 0.9	120	3.96 ± 0.5

The concentration of FGFC1 for the FGFC1 + EACA, FGFC1 + TXA, and FGFC1 + SBTI assays was 0.12 mM.

**Table 2:** Residues involved in the docking of EACA and FGFC1 to KR1–KR5 of plasminogen.

Domain	EACA		FGFC1	
	Residues in Hydrophobic Interactions	Residues in Hydrophilic Interactions	Residues in Hydrophobic Interactions	Residues in Hydrophilic Interactions
KR1	Asp55, Asp57, Tyr64, Tyr72, Trp62	Arg71, Arg35	Pro58, Pro31, Trp62, Asp55, Arg35, Arg33, Tyr72, Lys70, Glu69	His32, Arg71, Tyr64, Tyr74, Pro68, Asp57
KR2	Asp54, Asp56, Trp61, Trp71, Phe63	Arg70	Trp71, Asp54, Tyr35, Lys43, Pro53, Asn52, Glu7, Asn42, Phe40, Asp56, Trp61	Lys39, Asn55, Gly34
KR3	Arg36, His33, His64, Trp72, Trp62	Arg71, Lys57	Asp81, Leu2, Val17, Lys76, Tyr74, Cys75, Glu73, Trp72, Ala60, Arg59, Thr5	Ser79, Gly4, Ile 77
KR4	Trp125, Trp135, Asp119, Asp121, Phe127	Lys100, Arg134	Ser92, Ser91, Pro95, Thr129, Cys87, Lys86, Asn113, Glu103, Met112, Met93, Gln88, Thr111, Thr101, Pro102	Thr94, Ser89, Leu110
KR5	Tyr72, Tyr74, Tyr64, Asp55, Asp57, Trp62, Phe36	-	Asp57, Asp55, Tyr64, Tyr72, Leu71, Ile35, Trp62, Phe36, Thr40, Ser34	Asn41, Arg32

**Table 3:** The binding affinity values of the most appropriate binding modes obtained from AutoDock.

Ligand	Receptor	Binding Affinity (kcal/mol)
EACA	KR1	−5.2
EACA	KR2	−4.3
EACA	KR3	−3.7
EACA	KR4	−4.5
EACA	KR5	−4.3
FGFC1	KR1	−7.4
FGFC1	KR2	−9.0
FGFC1	KR3	−6.3
FGFC1	KR4	−8.3
FGFC1	KR5	−6.7

Note: The docking scoring function of AutoDock Vina worked well to rank binding poses. However, it is not necessarily related to the binding affinity between ligand and receptor.

## Data Availability

Data presented in this study are not available from the authors.

## Sample Availability

Samples of the compounds are not available from the authors.
